# Recurrent Spontaneous Hypothermia in an Elderly Woman: A Rare Neurological Case of Late-Onset Shapiro Syndrome

**DOI:** 10.7759/cureus.89920

**Published:** 2025-08-12

**Authors:** Aye Thinzar Moe, Sujata Lama, Thi Han, Kyaw Soe Tun

**Affiliations:** 1 General Medicine, King's College Hospital NHS Foundation Trust, London, GBR; 2 Geriatrics, King's College Hospital NHS Foundation Trust, London, GBR

**Keywords:** acute delirium, agenesis of the corpus callosum, elderly people, hypothermia, shapiro syndrome

## Abstract

An 81-year-old woman with a known diagnosis of Shapiro syndrome presented with recurrent hypothermia and delirium. This diagnosis had previously been made following multiple hospital admissions, extensive investigations, and imaging that showed corpus callosum agenesis. Shapiro syndrome is a rare neurological disorder characterised by episodic hypothermia and autonomic dysfunction. Management in this case was guided by an individualised care plan and supportive measures. This report highlights the diagnostic complexity of Shapiro syndrome and the importance of clinical awareness in older adults with unexplained hypothermia.

## Introduction

Shapiro syndrome is a rare neurological disorder characterised by the triad of spontaneous periodic hypothermia, hyperhidrosis and agenesis or dysgenesis of corpus callosum [1,2]. As of 2024, fewer than 100 cases have been reported in the literature to date [3]. It affects both genders, with a slight male predominance, and most often presents in childhood or adolescence, though late-onset cases are increasingly recognised [1,4,5]. The condition typically presents with recurrent episodes of hypothermia with diaphoresis or altered mental status. In older patients, episodes may present as delirium, lethargy, or unresponsiveness. The exact pathophysiology remains unclear, though hypothalamic dysregulation is the most widely accepted mechanism [1,3,4]. This case study adds to the limited literature on late-onset Shapiro syndrome, aiming to raise awareness in elderly patients, highlight the role of neuroimaging and exclusion of common causes, and present a management approach to support timely diagnosis and improve outcomes.

## Case presentation

An 81-year-old woman was brought to the emergency department after being found unresponsive by her carers at her extra care housing facility. On arrival, her only abnormal sign was that she was hypothermic with a core temperature of 32.9 °C; other observations were within normal limits. She appeared drowsy but rousable, with a Glasgow Coma Scale score of 14/15. Carers reported increased agitation and cognitive decline over the preceding 48 hours.

Her care home team highlighted a known diagnosis of Shapiro syndrome and an established care plan, outlining risks of delirium during hypothermic episodes. The advice was for conservative rewarming with blankets, Bair Huggers and fluids, avoidance of paracetamol during hypothermic phases, and hospital admission if her temperature remained below 32-33 °C despite management or if delirium occurred. 

During this admission, her presentation was consistent with previous hypothermic episodes of her known Shapiro syndrome. Basic blood work was performed to exclude obvious acute causes, including septic workup, which was negative. She was managed with conservative rewarming using blankets, Bair Huggers, and fluids, in line with her care plan. Her temperature improved, delirium resolved, and she was discharged once stable.

Her diagnosis of Shapiro syndrome had been made in 2018, almost a year after she first began experiencing daily episodes of feeling unusually cold or hot, often self-managed with temperature-appropriate drinks and occasionally paracetamol when febrile. However, several episodes were severe enough to lead to hospital admissions due to marked hypothermia, with delirium and reduced consciousness. These episodes were consistent in presentation, typically involving core temperatures below 33 °C, confusion, agitation, and cognitive impairment, all of which resolved with rewarming.

Given the recurrent nature of these unexplained hypothermic episodes and associated neurocognitive changes, an extensive blood workup and neurological workup were undertaken to exclude structural, inflammatory, infectious, metabolic, endocrine, nutritional or neoplastic causes. Her laboratory tests, including full blood count, renal and liver function, thyroid profile, glucose, cortisol, inflammatory markers, and electrolytes, were all normal. Blood and urine cultures were negative, and there was no biochemical evidence of malnutrition. She had a computed tomography (CT) scan of her head, which initially showed a possible cholesteatoma, later ruled out by the Otolaryngology team. CT chest, abdomen and pelvis excluded any primary malignancy or possible metastases. Her cerebrospinal fluid analysis on lumbar puncture, performed to exclude central nervous system infection or inflammation, was grossly normal, and all her autoimmune panels were unremarkable.

**Figure 1:**
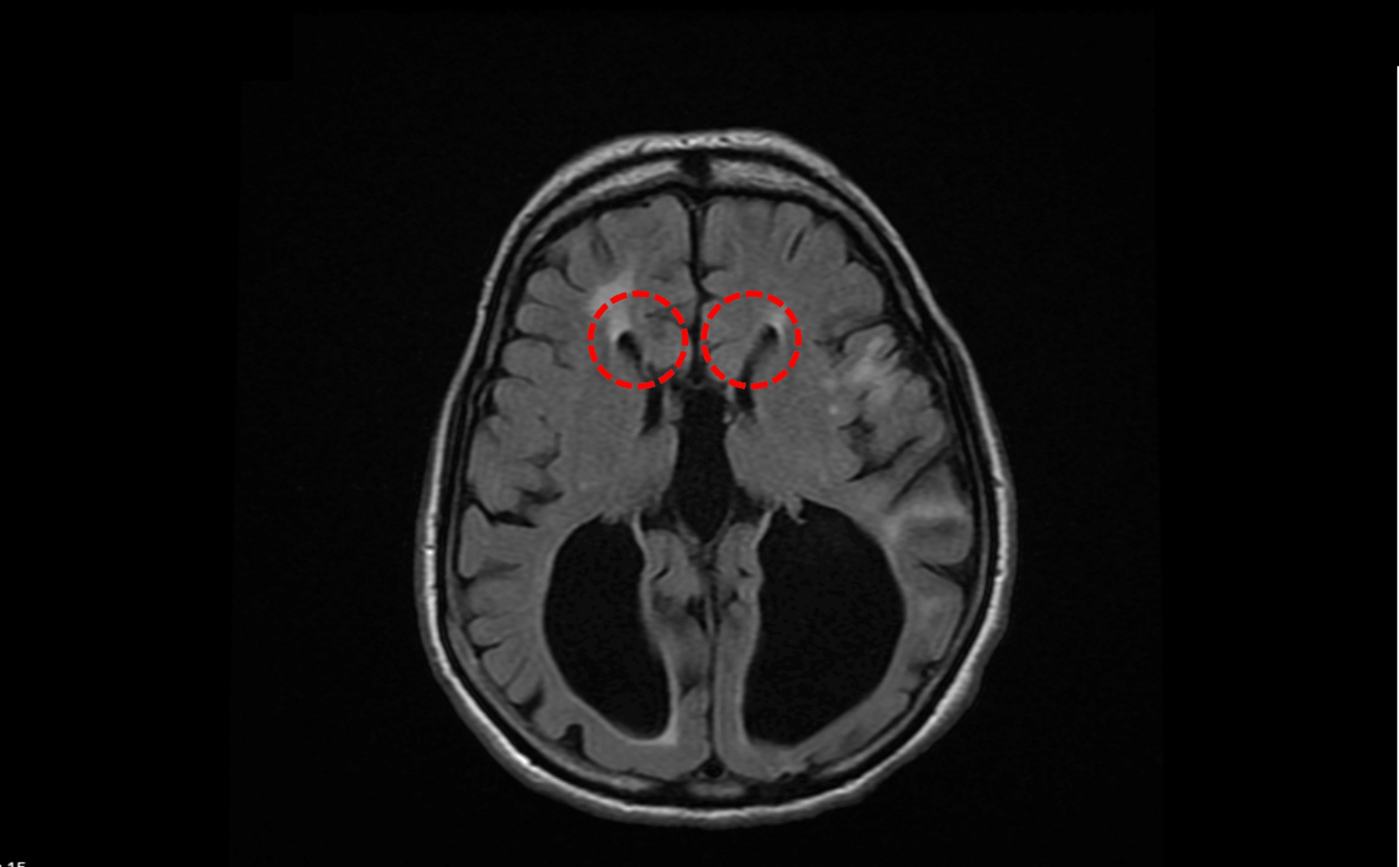
Coronal view of MRI brain showing a "moose head" appearance and enlargement of the lateral ventricles (red circle) due to agenesis of the corpus callosum MRI - Magnetic Resonance Imaging

**Figure 2:**
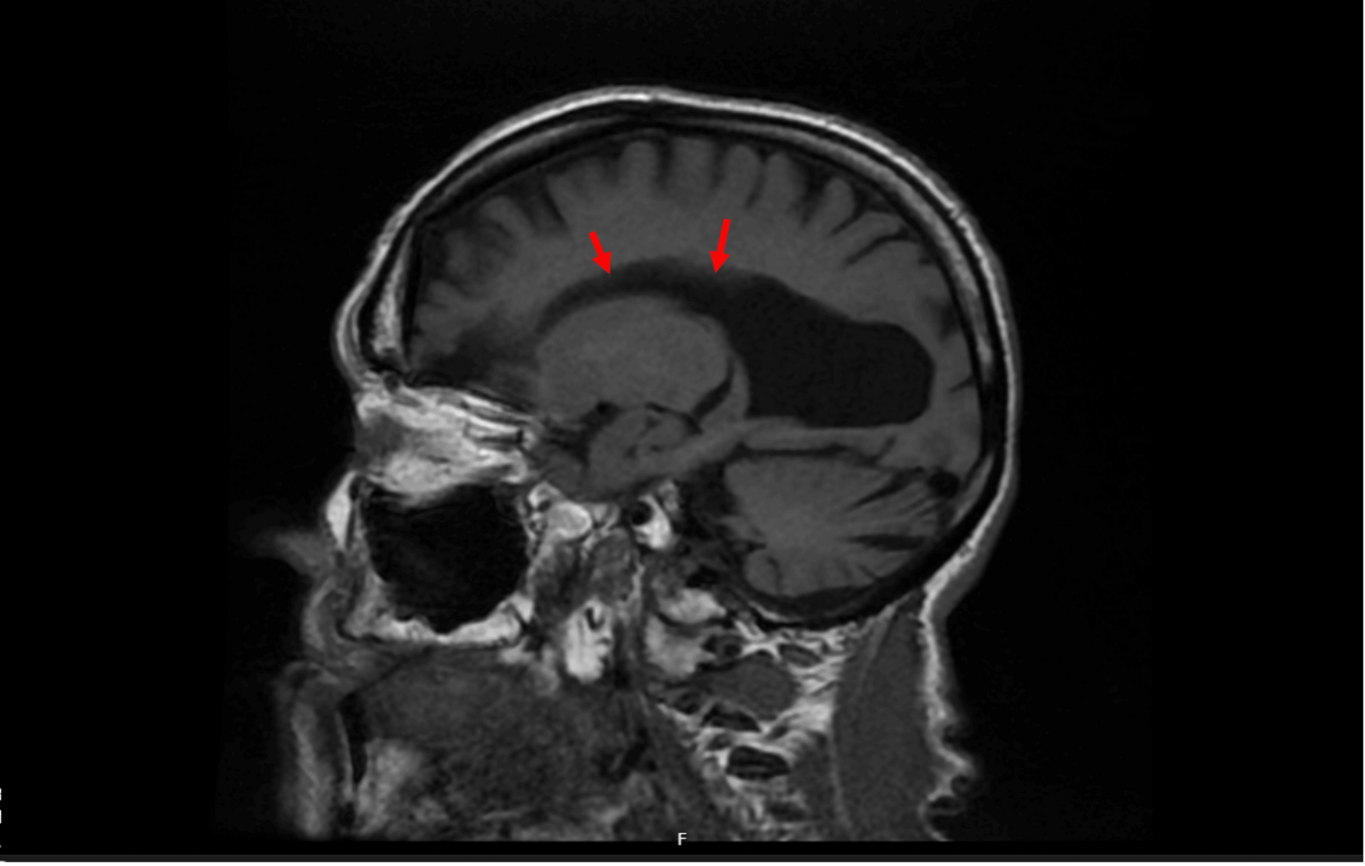
Sagittal view of MRI brain showing absence of the corpus callosum (red arrows) MRI - Magnetic Resonance Imaging

Crucially, her brain magnetic resonance imaging (MRI) revealed congenital agenesis of the corpus callosum along with significant volume loss likely related to the severe traumatic brain injury she had sustained in her 30s (Figures 1, 2). She went into a coma for several weeks during this time and subsequently developed post-traumatic epilepsy, though she made functional recovery and returned to work later.

Further evaluation with prolonged inpatient electro-encephalogram (EEG) and sleep studies demonstrated interictal abnormalities over the right anterior and left temporal regions, consistent with her known post-traumatic and vascular lesions. Occasional epileptiform discharges suggested a predisposition to focal seizures, but no autonomic events were recorded during the monitoring period. Sleep macrostructure was within normal limits.

Despite the absence of captured episodes during EEG, the combination of clinical presentation, characteristic corpus callosum agenesis on imaging, and exclusion of other causes led to the eventual diagnosis of Shapiro syndrome, a rare and often elusive explanation for her recurrent hypothermia and neurocognitive symptoms.

Following diagnosis, the patient was started on clonidine 25 micrograms once daily, which was gradually titrated to 50 micrograms three times daily. Since initiating this regimen, she has remained clinically stable, with a marked reduction in the frequency and severity of hypothermic episodes.

## Discussion

Shapiro syndrome is a rare neurological condition, first described in 1969, characterised by spontaneous recurrent hypothermia, and in most cases, agenesis or dysgenesis of the corpus callosum [2]. Because of its rarity and non-specific presentation, diagnosis is often delayed, particularly in older adults, where more common causes of hypothermia (e.g., infection, hypothyroidism, environmental exposure) are typically considered first [1,5].

The pathophysiology remains poorly understood. Shapiro originally hypothesised a form of ‘’diencephalic epilepsy’’ but this has been discounted, particularly considering non-epileptiform EEG findings and recognition of variant forms without callosal abnormalities [2,6]. More recently, three main hypotheses have emerged [1].

The most accepted theory involves hypothalamic dysfunction, particularly affecting the anterior and posterior nuclei that regulate heat dissipation and conservation [1,7,8]. A second theory suggests neurotransmitter imbalance, especially involving serotoninergic and dopaminergic pathways, which may explain the partial therapeutic response to agents such as cyproheptadine and clonidine [9-11]. A third, less established hypothesis proposes hypermelatoninemia, with isolated cases showing elevated melatonin levels during hypothermic episodes [12]. While these mechanisms are not mutually exclusive, hypothalamic dysfunction is generally regarded as the core pathological process [1,13].

Importantly, while callosal agenesis is common, variant forms with normal neuroimaging have been reported, so the diagnosis should not be excluded on imaging alone. In such cases, clinical features and systematic exclusion of alternative aetiologies are paramount [1,14].

Treatment is largely supportive. Central alpha agonists, targeting thermoregulatory pathways, such as clonidine and cyproheptadine, have been used with mixed success, although evidence remains limited [10,11]. Individual care plans are essential, outlining specific thresholds for intervention, avoidance of antipyretics during hypothermic phases, and clear escalation criteria, thereby reducing unnecessary treatments and enabling early recognition of clinical deterioration [1,14]. 

This case illustrates the diagnostic complexity of Shapiro syndrome in the elderly and highlights the importance of neuroimaging and longitudinal clinical context when evaluating recurrent, unexplained hypothermia, and the exclusion of more common aetiologies remains central to diagnosis.

## Conclusions

This case highlights the diagnostic complexity of Shapiro syndrome, particularly in older patients, where atypical presentation may lead to delayed recognition. For recurrent, unexplained episodes of hypothermia, especially when routine investigations are inconclusive, it is important for clinicians to consider rare neurological causes, including Shapiro syndrome, which remains a diagnosis of exclusion. Neuroimaging is essential to identify structural brain abnormalities, most notably involving the agenesis or dysgenesis of the corpus callosum. Although treatment is primarily supportive, central alpha-agonists like clonidine may offer symptomatic relief in select cases. Ultimately, a high index of clinical suspicion, careful exclusion of more common aetiologies and integration of clinical findings with brain imaging are essential for timely diagnosis and effective management of this rare but important syndrome.
